# Supplementary material for: Critical review of multimorbidity outcome measures suitable for low-income and middle-income country settings: perspectives from the Global Alliance for Chronic Diseases (GACD) researchers
Source: BMJ Open. 2020 Sep 6;10(9):e037079. doi: 10.1136/bmjopen-2020-037079 (PMC7478040; doi:10.1136/bmjopen-2020-037079)
Supplement: Author's manuscript [file bmjopen-2020-037079.draft_revisions.pdf]

BMJ Open is committed to open peer review. As part of this commitment we make the peer review history of every article we publish publicly available.

When an article is published we post the peer reviewers' comments and the authors' responses online. We also post the versions of the paper that were used during peer review. These are the versions that the peer review comments apply to.

The versions of the paper that follow are the versions that were submitted during the peer review process. They are not the versions of record or the final published versions. They should not be cited or distributed as the published version of this manuscript.

BMJ Open is an open access journal and the full, final, typeset and author-corrected version of record of the manuscript is available on our site with no access controls, subscription charges or pay-per-view fees (http://bmjopen.bmj.com).

If you have any questions on BMJ Open's open peer review process please email info.bmjopen@bmj.com

BMJ Open

A Critical Review of Multi-Morbidity Outcome Measures suitable for Low- and Middle Income Country Settings: perspectives from the Global Alliance for Chronic Diseases (GACD) Researchers

Journal:	*BMJ Open*
Manuscript ID	bmjopen-2020-037079
Article Type:	Original research
Date Submitted by the Author:	17-Jan-2020

Complete List of Authors:	Hurst, John; UCL Medical School,, Academic Unit of Respiratory Medicine<br>Agarwal, Gina; McMaster University, Family Medicine<br>van Boven, Job F. M.; Univ Groningen<br>Daivadanam, Meena; Karolinska Institutet, Department of Public Health Sciences; Uppsala University , Department of Food, Nutrition and Dietetics<br>Gould, Gillian; The University of Newcastle, School of Medicine and Public Health; University of Newcastle Hunter Medical Research Institute,<br>Wan-Chun Huang, Erick ; Woolcock Institute of Medical Research, Sydney, Australia<br>Maulik, Pallab; The George Institute for Global Health,<br>Miranda, J. Jaime; Universidad Peruana Cayetano Heredia, CRONICAS Centre of Excellence in Chronic Diseases<br>Owolabi, M. O.; University of Ibadan College of Medicine, Medicine<br>Premji, Shahirose ; York University,<br>Soriano, Joan; Universidad Autónoma de Madrid,<br>Vedanthan, Rajesh; NYU Langone Health,<br>Yan, Lijing; Global Health Research Center, Duke Kunshan University, Kunshan, Jiangsu, China<br>Levitt, Naomi; University of Cape Town , medicine

Keywords:	PRIMARY CARE, PUBLIC HEALTH, STATISTICS & RESEARCH METHODS, Clinical trials < THERAPEUTICS

SCHOLARONE™
Manuscripts

BMJ

I, the Submitting Author has the right to grant and does grant on behalf of all authors of the Work (as defined in the below author licence), an exclusive licence and/or a non-exclusive licence for contributions from authors who are: i) UK Crown employees; ii) where BMJ has agreed a CC-BY licence shall apply, and/or iii) in accordance with the terms applicable for US Federal Government officers or employees acting as part of their official duties; on a worldwide, perpetual, irrevocable, royalty-free basis to BMJ Publishing Group Ltd ("BMJ") its licensees and where the relevant Journal is co-owned by BMJ to the co-owners of the Journal, to publish the Work in this journal and any other BMJ products and to exploit all rights, as set out in our licence.

The Submitting Author accepts and understands that any supply made under these terms is made by BMJ to the Submitting Author unless you are acting as an employee on behalf of your employer or a postgraduate student of an affiliated institution which is paying any applicable article publishing charge ("APC") for Open Access articles. Where the Submitting Author wishes to make the Work available on an Open Access basis (and intends to pay the relevant APC), the terms of reuse of such Open Access shall be governed by a Creative Commons licence – details of these licences and which Creative Commons licence will apply to this Work are set out in our licence referred to above.

Other than as permitted in any relevant BMJ Author's Self Archiving Policies, I confirm this Work has not been accepted for publication elsewhere, is not being considered for publication elsewhere and does not duplicate material already published. I confirm all authors consent to publication of this Work and authorise the granting of this licence.

For peer review only

A Critical Review of Multi-Morbidity Outcome Measures suitable for Low- and Middle Income Country Settings: perspectives from the Global Alliance for Chronic Diseases (GACD) Researchers

John R Hurst, Gina Agarwal, Job F M van Boven, Meena Daivadanam, Gillian S Gould, Erick Wan-Chun Huang, Pallab K Maulik, J Jaime Miranda, Mayowa O Owolabi, Shahirose Sadrudin Premji, Joan B Soriano, Rajesh Vedanthan, Lijing L Yan, Naomi S Levitt *

***: on behalf of the GACD Multi-Morbidity Working Group contributors:**
Ricardo Araya, Kirsten Bobrow, Niels H Chavannes, F Xavier Gómez-Olivé, Shabbar Jaffar, Bruce J Kirenga, Rianne M J J van der Kleij, Muralidhar M Kulkarni, Laura Loli-Dano, Patricio Lopez-Jaramillo, Shane Norris, Josefien van Olmen, Gary Parker, Trishul Siddharthan, Kamran Siddiqi, Najma Siddiqi, Antigona C Trofor

Corresponding Author:
John R Hurst
Professor of Respiratory Medicine
UCL Respiratory
University College London
London
UK

j.hurst@ucl.ac.uk

For peer review only

Funding
The article was supported by the Global Alliance for Chronic Diseases (GACD) secretariat. The Authors are investigators on individual studies funded in collaboration with the GACD.

Conflict of Interest
The Authors have no conflicts of interest to declare in relation to this work.

KEYWORDS: Multimorbidity; Outcome Assessment, Health Care;

WORDCOUNT: 4672

Abstract

OBJECTIVES: There is growing recognition around the importance of multi-morbidity in low- and middle-income country (LMIC) settings, and specifically the need for pragmatic intervention studies to reduce the risk of developing multi-morbidity, and of mitigating the complications and progression of multi-morbidity in LMICs. One of many challenges in completing such research has been the selection of appropriate outcomes measures. A 2018 Delphi exercise to develop a core-outcome set for multi-morbidity research (COSmm) did not specifically address the challenges of multi-morbidity in LMICs where the global burden is greatest, patterns of disease often differ and health systems are frequently fragmented. We therefore aimed to summarise and critically review outcome measures suitable for studies investigating mitigation of multi-morbidity in low- and middle-income country (LMIC) settings..

SETTING: LMIC

PARTICIPANTS: people with multi-morbidity.

OUTCOME MEASURES: identification of all outcome measures

RESULTS: We present a critical review of outcome measures across eight domains: mortality, quality of life, function, health economics, health-care access and utilization, treatment burden, measures of 'healthy living', and self-efficacy and social functioning.

CONCLUSIONS: Studies in multi-morbidity are necessarily diverse and thus different outcome measures will be appropriate for different study designs. Presenting the diversity of outcome measures across domains should provide a useful summary for researchers, encourage the use of multiple domains in multi-morbidity research, and provoke debate and progress in the field

TRIAL REGISTRATION: Not applicable.

Strengths and Limitations

- There is no existing review of outcome measures suitable for use in studies to mitigate multi-morbidity in LMIC settings.
- The article is the written by the Global Alliance for Chronic Diseases researchers.
- It is not a systematic review.
- Further work is required to develop a core-outcome set for use in LMIC.

For peer review only - http://bmjopen.bmj.com/site/about/guidelines.xhtml

Introduction

There is growing recognition around the importance of multi-morbidity in low- and middle-income countries (LMICs) [1]. Multi-morbidity, as defined by the United Kingdom Academy of Medical Sciences (AMS) refers to "*the co-existence of two or more chronic conditions, each of which is either a physical non-communicable disease of long duration, a mental health condition of long duration, or an infectious disease of long duration*" [1]. The AMS report highlights challenges in delivering multi-morbidity research [2], including the selection of appropriate outcome measures. In 2018, Smith completed a Delphi exercise to develop a core-outcomes set for multi-morbidity research (COSmm) [3]. The highest scoring outcomes were health-related quality of life, mental health outcomes and mortality. Whilst ground-breaking, this process did not specifically target the challenges of multi-morbidity in LMICs where the global burden is greatest, patterns of disease often differ and health systems are frequently fragmented.

The Global Alliance for Chronic Diseases (GACD) is an alliance of health-research funders, whose research teams form a network of multidisciplinary researchers from both LMICs and high-income countries (HICs). We aim to reduce the impact of non-communicable diseases (NCDs) through a focus on implementation science research in LMICs, and high-priority populations in HICs. Recognizing synergies across our disease-specific programmes, in 2017 we formed a Multi-Morbidity Working Group and published a GACD Researchers' Statement concluding that "*a greater focus on multi-morbidity is overdue and necessary to successfully improve global health outcomes*", thus acknowledging the specific challenge of multi-morbidity in the LMIC context [4]. The statement went on to propose three strategic objectives, one of which was to change the way research is commissioned, funded and delivered when considering NCDs in LMICs.

Discussion with research funders subsequently highlighted that one barrier to funding research addressing multi-morbidity in LMICs was a perceived lack of robust outcome measures. We have therefore developed this GACD Researchers' perspective on outcome measures suitable for studies of multi-morbidity in LMICs, taking into account the challenges of (routine) data collection, and patient-provider factors such as differences in interpreting social constructs and health literacy. The intent is to build on the COSmm work [3]. Derived from a common base of expertise in NCD implementation research in LMICs, we present a diversity of potential measures that can accommodate different aspects of impact in LMICs, ranging from individual level outcomes to health service and health system effects. This is not an attempt to provide a core outcome measures set. Rather, together, the potential outcome measures inform different evaluations of effectiveness and/or process for multi-morbidity. We present these as a useful resource for those designing and reviewing intervention studies for multi-morbidity in LMIC settings, and hope this initiative may promote harmonization across studies that will be essential to better map the impact of multi-morbidity in LMIC settings.

For peer review only - http://bmjopen.bmj.com/site/about/guidelines.xhtml

Method

Potential outcome measures suitable for studies of multi-morbidity in LMICs were collected through a survey among the GACD multi-morbidity working group, and distilled by the writing committee (the Authors) into categories. All measures had to be suitable for use in multi-morbidity intervention studies in LMIC, either at the individual or the population level, and from an implementation science perspective. Criteria for suitability included ease of measurement, generalizability and statistical considerations. Each outcome approach is fully described below. The initial synthesis was reviewed by members of the GACD Multi-Morbidity Working Group for additional comments and suggestions (the Contributors). The resulting narrative review summarizes the group's collective thoughts within each domain of outcome measures studied.

Patient and Public Involvement

Patients or the public were not involved in the design, conduct, reporting, or dissemination plans of our research.

Outcome Measures for Multi-Morbidity Interventions in LMIC

1. Mortality

Death is the final common outcome for all individuals. Thus (premature) mortality is the most broadly applicable, generalizable, and comparable outcome for multi-morbidity research. Indeed, mortality was considered as an "essential" core outcome measure for multi-morbidity research according to the COSmm consensus [3].

However, precisely because mortality is so broadly applicable, it suffers from a lack of specificity. While cause-specific mortality is a potential solution to the issue of specificity, this approach moves away from the goal of multi-morbidity-based outcome consideration. In addition, mortality does not reflect the quality of life that an individual experiences during the time of survival; particularly in the context of multi-morbidity, both disability and quality-of-life considerations are important in terms of an individual's experience of illness, wellness and life. Indeed, death is not always the most important outcome from a patient-centered perspective, as has been demonstrated in studies assessing patient preferences of different potential health outcomes [5-7] and conceptualized as Disability-Adjusted Life Years.

Practical challenges with mortality as an outcome measure include statistical power and sample size for an outcome that is relatively rare compared to other outcomes and proxies, potentially requiring much longer follow-up periods, except for older and/or more severely affected populations. It is, however, generally easy to measure and while the primary cause may be ascertained through techniques such as verbal autopsy (2016 WHO VA standard) [8], assessing the contribution of multi-morbidity at verbal autopsy is more challenging. Whilst misclassifying the cause of death can impact the effect size for cause-specific mortality, power will be preserved for all-cause mortality. In some LMICs, ascertainment of deaths remains difficult due to the lack of mature vital registry systems and cultural traditions promoting deaths at home with delay in reporting.

For peer review only - http://bmjopen.bmj.com/site/about/guidelines.xhtml

Thus, mortality as an outcome for multi-morbidity research has been infrequently utilized, particularly in the context of LMIC settings [9-11]. Demographic surveillance sites that have a long record of verbal autopsy could, however, provide a useful data reservoir to examine associations between multi-morbidity and mortality

2. Generic Quality of Life scales

Health-related quality of life (HRQoL) instruments measure multidimensional wellbeing and functioning. Such scales may be generic such as EQ-5D and SF-36, or disease (/area) specific. While disease-specific measures may have better content and face validity as well as better responsiveness and sensitivity to change compared to generic measures, generic measures are (by definition) not disease specific and likely better for comparison of HRQoL among different diseases and for diseases in combination, an important consideration for multi-morbidity research.

Among generic tools, the COSmm consensus [3] ranked the EQ-5D, SF36 and ’12, and Global quality of life (WHOQOL-BREF) most highly
.
The EQ-5D [12] has been widely used since introduction in the 1990s, facilitating health-economic analysis (see below). It is designed to be completed by the participant and is available in multiple languages and thus widely applicable. The EQ-5D questionnaire has two components (health-state description and evaluation). In the health state description, health status is measured across five dimensions: mobility, self-care, usual activities, pain/discomfort, and anxiety/depression. In the evaluation section, the respondents evaluate their overall health status using a visual analogue scale.

The SF-36 [13] has 36 questions across eight domains: vitality, physical functioning, bodily pain, general health perceptions, physical role functioning, emotional role functioning, social role functioning and mental health.

The WHOQOL-BREF [14] is an abbreviated version of the WHOQOL-100 quality of life assessment, originally developed by the WHOQOL Group working across fifteen international field centers to develop a quality of life assessment applicable across multiple settings.

HRQoL tools have a number of advantages over mortality as an outcome, being amenable to changes in the short term. HRQoL outcomes are particularly meaningful as the aim of clinical treatment and management is generally optimizing quality of life. Consequently, managing multi-morbidity needs to take quality of life into account both as an outcome marker, but also an input factor into formulating clinical management. Practical considerations in LMIC include the availability of valid translations in local languages, and the challenges of use in populations with low literacy or understanding of visual-analogue scales. Other unanswered questions include whether thresholds for minimum clinically important differences on these scales should be altered in the context of multi-morbidity. Notably, some common NCDs such as hypertension are not generally associated with significant symptom burden.

For peer review only - http://bmjopen.bmj.com/site/about/guidelines.xhtml

3. Multi-dimensional indices of function

The AMS [1] recommended that reports of multi-morbidity should provide details of functional deficits, or disabilities and frailty. In both instances the recommendation was made that this should be coded using a standardized classification scheme. For the former, the WHO Disability Assessment Schedule 2.0 (WHODAS 2.0) or the International Classification of Functioning, Disability and Health (ICF) were suggested. For the latter, the cumulative deficit model of frailty or Fried's phenotype model was recommended (see below).

WHO Disability Assessment Schedule

The WHODAS 2.0 has been widely used in epidemiological and observational studies in LMICs. It is a self-administered 12 item questionnaire that assesses six different adult life tasks over the preceding month. The specific areas covered are 1) understanding and communication; 2) self-care; 3) mobility; 4) interpersonal relationships; 5) work and household roles and 6) community and civic roles. WHODAS has been included as a secondary outcome measure in three multi-morbidity trials in LMIC (currently unreported [15-17]).

Frailty assessment instruments:

There are many methods to assess frailty including the Fried Index, the Frailty Index and the British Frailty Index. While these have been used to examine the prevalence, correlates or outcomes of frailty in LMIC, further validation is still required in these settings [18]. Of the various metrics, the Fried Index [19] has been the most commonly used in LMIC. This index measures frailty by the presence of three or more of five physical deficits - exhaustion, weakness, slowness, low levels of activity and weight loss. Three of the items are collected using questionnaires, but slowness is assessed using a walking test and weakness by assessing grip strength. The Frailty Index has also been commonly used in LMICs and uses the presence or absence of medical conditions or poor performance on functional tasks to assess the number of deficits present and thus frailty [18]. Using frailty as an outcome measure for intervention studies in patients with multi-morbidity in LMIC is limited by factors such as a lack of equipment, and the question remains as to how susceptible to change such measurements are, and what a minimum clinically important difference (MCID) might be. Despite this, frailty instruments remain an important outcome in LMIC settings given that frailty may be a confounding factor in self-care, treatment adherence and family burden.

Assessment of physical functioning:

Physical functioning measures are commonly studied outcomes. The most frequently used indices include activities of daily living (such as eating, dressing and toileting), instrumental activities of daily living (such as shopping and answering phone calls), and the Barthel Index (self-reported outcomes on degree of assistance needed for mobility, self-care and continence). Smith [3] described activities of daily living, physical function and physical activity as core outcomes in multi-morbidity interventions. For ADL the following measures received greatest support: Frenchay Activities Index (FAI), Nottingham Extended Activities of Daily Living (NEADL) and the Instructions for Activities of Daily Living questionnaire (ADL/ IADL), but these have not been evaluated in the context of LMICs.

For peer review only

For peer review only - http://bmjopen.bmj.com/site/about/guidelines.xhtml

The modified Rankin Scale is an example of a disease-specific (in this case, stroke) composite outcome measure including rating of functioning from no interference with daily life, through various degrees of disability to death. These outcomes are relatively easy to assess and have particular relevance in LMICs as people generally express strong desires in maintaining physical functioning including their ability to work, avoiding financial consequences and burden on family caregiving.

4. Health Economic indices

The AMS report [1] highlighted the economic burden of multi-morbidity in LMICs and thus health economic indices are a rational choice as multi-morbidity outcome measures. However, most economic data on multi-morbidity were gathered in HICs and the question arises as to whether measurement instruments, data and outcomes commonly used to assess cost implications of multi-morbidity in HICs are applicable to LMIC settings.

One of the most common economic evaluations of healthcare interventions makes use of a technique called cost-effectiveness analysis and specifically the *incremental cost-effectiveness ratio* (ICER) [20]. The method to calculate the ICER is not disease specific, making it just as suitable to assess multi-morbidity interventions as single disease interventions. However, it requires specific attention to the definitions and collection of costs and effect data in LMICs. Within this ratio, costs and effects can be defined, measured, and calculated in different ways, of which some are more suitable in economic assessment of multi-morbidity interventions in LMICs than others. Interpretation of the ratio may differ in different settings.

In healthcare, interventions can impact different types of direct and indirect costs within and outside healthcare systems. The different costs to be included in cost-effectiveness analysis depends on the perspective that is taken (e.g. the healthcare payer, the society, the patient, or the family). Costs that directly result from the intervention and that which occur within healthcare systems should be included when a healthcare payer perspective is taken. However, in LMICs that lack universal health coverage, the perspective of the patient and family may be more relevant and a key focus could be on out-of-pocket costs. Examples of indirect costs are work productivity losses and these costs are especially relevant when a patient or societal perspective is taken.

In health economic studies, the effect of intervention uses a measure that is independent of a specific disease: the quality-adjusted life year (QALY). The QALY is a combination of utility (preferably measured using the EQ-5D) and survival. With the EQ-5D, certain health states are defined, to which a specific utility is assigned. Utility is the value a society gives to a specified health state and for each country a specific algorithm should be estimated from large general population samples. In many LMIC settings these still need to be further developed to allow for generalizable models of effectiveness.

While most HICs have defined guidelines and make use of fixed thresholds or ranges to assess whether a certain ICER is considered cost-effective, such guidelines and thresholds are

For peer review only - http://bmjopen.bmj.com/site/about/guidelines.xhtml

generally lacking in LMICs. This complicates the interpretation of cost-effectiveness analyses in LMICs. As a general rule, the World Health Organization (WHO) defines an intervention that costs less than three times the gross domestic product per capita as cost-effective [21]. It is important to note that the economic analyses discussed here are not specific to multi-morbidity, but are nonetheless suitable for the study of multi-morbidity.

5. Health Care Access and Utilization

Multi-morbidity is associated with repeated care seeking, often at different providers. This not only results in multiple interactions with health care settings through outpatient and inpatient admissions but also involves para-medical services and practitioners of traditional medicine.

Although we identified no study that has specifically looked at generating or testing multi-morbidity related healthcare access indices in LMICs, the WHO Study on Global Ageing and Adult Health (SAGE) which focused on LMICs tracked indicators specific to multi-morbidity in ageing populations [22, 34]. These included the number of outpatient visits in the last 12 months, overnight hospital stays in the past three years, and the number of overnight stays in hospital in the past 12 months. A UK National Health Service document [24] outlines equity indicators that may also map multi-morbidity relevant in LMIC settings, and some of these have direct healthcare access relevance such as emergency hospitalizations for chronic conditions and repeat emergency hospitalizations in the same year. Access to medicines listed on the WHO Essential Medications list would provide another metric, as would recommendations on attention to comorbidity and pharmacological interactions in treatment guidelines.

This lack of LMIC specific multi-morbidity indices to plot healthcare access leads to a critically important avenue of research that could draw on that conducted in HICs [25]. The latter work lists a range of objectives that need to be addressed in healthcare practices catering to clients with multi-morbidity and lists a set of preventive services for such cases which health facilities should provide. Health-seeking behaviour is a further dimension related to healthcare access that is shaped by unique socioeconomic and cultural contexts faced by patients in LMICs. We suggest it would be useful to develop health-seeking behaviour indices relevant across LMICs. This needs a contextual framework to best understand what is feasible and what can be tracked within specific LMIC settings, acknowledging the challenges introduced by the fragmentation of care and the multiplicity of levels of provision of care in the public and private sectors. Such indices could be linked with existing monitoring frameworks used to assess Universal Health Coverage [26].

The Global Burden of Disease initiative has recently incorporated a new metric at national level termed the Healthcare Access and Quality (HAQ) Index [27]. The HAQ index is a scale from 0 to 100, calculated by measuring mortality rates from causes that should not be fatal (amenable mortality) in the presence of effective medical care. This correlates with the Socio-demographic Index, a measure of overall development consisting of income *per capita*, average years of education, and total fertility rates.

For peer review only - http://bmjopen.bmj.com/site/about/guidelines.xhtml

6. Treatment burden

The burden of treatment, a relatively new concept, emerged from disease-centered healthcare systems in response to the growing needs of coping with chronic conditions. In the context of multi-morbidity, this may be considered as the workload and impact on a patient as a result of receiving medical care [28]. High treatment burden may lead to overwhelmed patients who struggle to access healthcare and adhere to suggested treatment whilst coordinating their own care and other aspects of life, a particular issue among patients with multi-morbidity. As a consequence, polypharmacy and non-adherence to treatment and poor clinical outcomes may follow, resulting in an even higher burden of treatment, a deterioration cycle depicted in the Cumulative Complexity Model [29]. Therefore, assessing treatment burden is a priority in order to achieve better quality healthcare, and treatment burden is a potential outcome measure in interventions directed against multi-morbidity. There is also the challenge, more pronounced in LMICs, that in areas of no care there can be no 'burden' from treatment which it is impossible to access.

Assessing the burden of treatment is not an easy task. It generally requires multi-dimensional measures that are tailored to the medical condition(s), health system(s) and cultural background. Tailoring to specific conditions may diminish value in multi-morbidity. Eton proposed a conceptual framework of treatment burden based on qualitative inquiries to patients with chronic conditions, consisting of three themes and 15 subthemes [30]. A number of tools for evaluating treatment burden for patients with multi-morbidity have been developed in the past few years. Eton designed and validated the Patient Experience with Treatment and Self-management (PETS) [31]. The Treatment Burden Questionnaire (TBQ) is another instrument, consisting of 15 items [32] and later further adapted [33, 34]. In 2018, Duncan published the Multimorbidity Treatment Burden Questionnaire (MTBQ), a ten-item measure initially validated in primary care in the United Kingdom [35]. The Healthcare Task Difficulty (HCTD) questionnaire is an 11-question tool designed to measure only one aspect – perceived difficulty in performing healthcare management tasks [36]. Finally, the Multi-morbidity Illness Perceptions Scale (MULTIPLes), unlike other instruments, was designed to measure the perceived impact of multi-morbidity [37]. The scale includes treatment burden (six questions) as one of the subscales.

As these questionnaires are relatively new, validation and translation for different populations and geographic areas remain limited, especially in LMICs. Exploring the notion and measurement of treatment burden in LMIC remains relatively unexplored [34, 38].

There are a number of remaining issues to be considered before applying these tools in LMICs. Firstly, the strengths and limitations of each tool should be examined as careful validation has often not been conducted in such settings. Secondly, using mixed methods may help identify relevant issues relating to differences in contexts, cultures and health system structures. Thirdly, as all of these instruments have been available for less than a decade, longitudinal evidence of change over time is absent.

For peer review only - http://bmjopen.bmj.com/site/about/guidelines.xhtml

7. Measures of 'Healthy Living'

Multi-morbidity is complex to operationalize, which makes common denominators very relevant. Measures of 'Healthy Living' are direct common denominators for being at risk of developing individual components of multi-morbidity, and thus measuring change in these measures provides potential generic outcomes of interventions to mitigate future multi-morbidity. Most current behavioral interventions have targeted only one behaviour at a time.

Healthy living encompasses many different aspects of health and wellbeing, including diet, physical activity including sedentary behaviour, tobacco and alcohol consumption, developing health literacy, maintaining good hygiene, and sanitation. Most current behavioral interventions have targeted only one behaviour at a time.

Diet: Dietary assessments are complex. Self-reported dietary intake measurements are the most common form of dietary assessments, which include prospective recording of actual food consumed or retrospective recall [39, 40]. With respect to multi-morbidity, the focus must be on long-term usual intake. Dietary diversity scores are one such measure that can be estimated for the individual, or the household using counts of food items (food variety score) or food groups (dietary diversity score) consumed over a pre-specified period [41, 42]. Dietary diversity can be estimated at the Household level using the Household Dietary Diversity Score (HDDS), which assesses household access to a variety of foods, or at individual level for women and children respectively using the Minimum Dietary Diversity for Women of Reproductive Age tool (MDD-W) and WHO Infant and Young Child Minimum Dietary Diversity Tool (IYCF-MDD) [43].

Physical activity (PA) including sedentary behaviour: Convincing interventional evidence showing a clear dose-response relationship between PA and improved health outcomes comes mainly from HICs, although associations of PA with reduced cardiovascular mortality and morbidity are available globally [44]. Sedentary behaviour, defined as those that involve sitting or reclining and low levels of energy expenditure during waking hours [45], has also been associated with having at least two morbidities, independent of light, moderate or vigorous PA [45, 46] in HICs and LMICs. The global physical activity questionnaire (GPAQ) that is part of the WHO STEPwise Approach to Chronic Disease Risk Factor Surveillance data collection tool [47] is a commonly used tool to collect self-reported data on PA. The GPAQ which is a shorter (16-item) version of the longer International Physical Activity questionnaire (IPAQ) also assesses sitting time in addition to PA in three domains (work, travel and leisure-time). This is used to estimate the duration of moderate to vigorous physical activity (MVPA) or intensity in terms of MET (metabolic equivalent)-minutes per week of total and domain-specific activities. However, agreement between PA estimated by GPAQ and more objective measures has been moderate at best. Objective measures of PA allow real-time monitoring and can be easily completed using an application on a mobile device or a wearable pedometer or accelerometer, although this has mostly been tested in HIC settings. Considering the rapid acceleration of smart phone ownership in LMIC, and the availability of cheaper but robust wearable devices, these are now viable options and an optional tool to capture objective PA has since been incorporated into the GPAQ.

For peer review only - http://bmjopen.bmj.com/site/about/guidelines.xhtml

Tobacco and alcohol use: Tobacco use has been consistently linked as a causative factor for chronic respiratory disorders such as chronic obstructive pulmonary disease, cardiovascular disease and many cancers including lung cancer. Similarly, alcohol use has strong associations with NCDs. Ever and current use of tobacco or current use of alcohol are commonly used assessments in addition to questions focusing on frequency and amount of consumption, and these are part of the WHO STEPS instrument [47]. Where available, verification of smoking status can be achieved through measurement of carbon monoxide or urinary cotinine. Assessment of household, environmental and occupational airborne exposures are more complex.

Healthy living index: In addition to individual risks and behaviors, composite indicators that assess healthy living may be more relevant in the context of multi-morbidity. Tools to assess the environment in terms of its potential to offer opportunities for healthy living have been limited, especially in LMICs. Environmental Profile of a Community's Health (EPOCH) is a quantitative tool designed to capture community perceptions of tobacco, nutrition, and social environments, validated in five countries (China, India, Brazil, Colombia, and Canada) [48, 49]. EPOCH comprises an objective assessment of the physical environment, and an interviewer-administered questionnaire on residents' perceptions of their community to capture both objective and subjective measures of the environment [48]. The Community Healthy Living Index (CHLI), developed in the US assessed the environmental support potential of a community across five domains assessing a specific venue: schools, afterschool child care sites, work sites, neighborhoods, and communities-at-large [50]. Such tools could be adapted for use in LMICs.

8. Self-efficacy and social functioning

Self-efficacy and social functioning relate to social determinants of health such as age, gender, marital status, family background, employment, education level and socioeconomic status [51-57], affecting in turn how an individual is able to look after their health conditions (self-efficacy) and interact in society with other individuals leading a fulfilling life (social functioning). This raises the important question of whether indices of self-efficacy and social functioning may be suitable as outcomes measures in studies to mitigate multi-morbidity in LMIC settings.

There are limited studies that explore which social determinants are more influential than others in determining self-efficacy and social functioning. Positive personality traits and higher self-esteem demonstrated in adolescence positively affect self-efficacy [58]. Competent behaviour, such as skills of focusing on others' well-being, affiliative behaviours/interpersonal cooperation and participation, which are culturally valued and socially competent are associated with higher self-efficacy [59-60]. Liebke and colleagues [61] reported that loneliness and social functioning are associated. Loneliness may be caused by impaired social skills, such as maintaining conversations or expressing feelings, which are essential to adequate social functioning [61]. Values placed on social determinants of health may vary across different cultures. Differences in cultural traditions may affect the sources of self-efficacy belief systems [51, 54, 55].

For peer review only - http://bmjopen.bmj.com/site/about/guidelines.xhtml

Given the multitude of cultural factors affecting the precursors of self-efficacy and social functioning, populations in LMICs may have fewer opportunities to develop such skills. Therefore, whilst measures of self-efficacy and social functioning could be used as multi-morbidity outcome measure in LMIC, a single index is unlikely to be useful across all settings.

Conclusions

The case has been made for the growing global importance of multi-morbidity, the need for pragmatic intervention studies to reduce the risk of developing multi-morbidity in LMIC settings, and of mitigating the complications and progression of multi-morbidity. One of many challenges in such research has been the selection of appropriate outcomes measures.

We present the GACD Researchers' perspective on outcome measures suitable for multi-morbidity intervention studies in the context of LMICs. We have considered outcome measures across eight domains (Figure 1). Some represent direct measurements of clinical outcomes, whilst others represent intermediate variables on the pathway to multi-morbidity. Some measures are single, others are composite. They vary in their ease of collection. It is critical to choose appropriate outcomes for the study design selected in order to demonstrate and understand the effect of an intervention. Studies in multi-morbidity are necessarily diverse and thus different outcome measures will be appropriate for different study designs. As with the COSmm consensus [3], we recognize the importance of mortality and health-related quality of life as multi-morbidity outcomes, and these are suitable for use in LMIC settings. Many other outcomes from the COSmm work, including patient-reported impacts and behaviors (such as treatment burden and self-efficacy); physical activity and function, and health systems indicators (notably health economic indices) are also suitable for LMIC settings, though in the context and with the caveats that we have described above.

The diversity of outcome measures across domains demonstrated here should provide a useful summary for researchers, and encourage the use of multiple domains in multi-morbidity research, rather than just a single outcome measure. Ultimately, the proof of utility for these outcome measures will be the demonstration that an effective multi-morbidity intervention can improve the health of the community in which it is tested. Meanwhile, there remains the urgent need for further study and development of outcome measures suitable for multi-morbidity intervention studies in the context of LMIC.

This work is not intended to be a core outcome set, nor a systematic review. Instead, we present a critical, narrative synthesis describing the range of outcome measures that might be selected for use in such settings, and their challenges. We anticipate this will be useful to other researchers designing and conducting such studies, and to provoke debate and progress in the field.

For peer review only

For peer review only - http://bmjopen.bmj.com/site/about/guidelines.xhtml

Figure Legend

FIGURE 1: *Eight domains of outcome measures for multi-morbidity interventions in LMIC.*

References

1. Academy of Medical Sciences. Multimorbidity: a priority for global health research: Full report. Available at https://acmedsci.ac.uk/policy/policy-projects/multimorbidity last accessed 1st August 2019.

2. Academy of Medical Sciences. Advancing research to tackle multimorbidity: the UK and LMIC perspectives workshop report. Available at https://acmedsci.ac.uk/policy/policy-projects/multimorbidity last accessed 1st August 2019.

3. Smith SM, Wallace E, Salisbury C, Sasseville M, Bayliss E, Fortin M. A Core Outcome Set for Multimorbidity Research (COSmm). Ann Fam Med. 2018 Mar;16(2):132-138

4. Hurst JR, Dickhaus J, Maulik PK, Miranda JJ, Pastakia SD, Soriano JB, Siddharthan T, Vedanthan R; GACD Multi-Morbidity Working Group. Global Alliance for Chronic Disease researchers' statement on multimorbidity. Lancet Glob Health. 2018 Dec;6(12):e1270-e1271.

5. Vaanholt MCW, Kok MM, von Birgelen C, Weernink MGM, van Til JA. Are component endpoints equal? A preference study into the practice of composite endpoints in clinical trials. Health Expect. 2018 Dec;21(6):1046-1055. doi: 10.1111/hex.12798. Epub 2018 Aug 14. PubMed PMID: 30109764; PubMed Central PMCID: PMC6250862

6. van Summeren JJ, Schuling J, Haaijer-Ruskamp FM, Denig P. Outcome prioritisation tool for medication review in older patients with multimorbidity: a pilot study in general practice. Br J Gen Pract. 2017 Jul;67(660):e501-e506. doi: 10.3399/bjgp17X690485. Epub 2017 Mar 27. PubMed PMID: 28347987; PubMed Central PMCID: PMC5565860

7. Fried TR, Tinetti M, Agostini J, Iannone L, Towle V. Health outcome prioritization to elicit preferences of older persons with multiple health conditions. Patient Educ Couns. 2011 May;83(2):278-82. doi: 10.1016/j.pec.2010.04.032. Epub 2010 May 31. PubMed PMID: 20570078; PubMed Central PMCID: PMC2945432

8. WHO. Verbal autopsy standards: ascertaining and attributing causes of death. Available at https://www.who.int/healthinfo/statistics/verbalautopsystandards/en/ last accessed 1st August 2019.

9. Garvey J, Connolly D, Boland F, Smith SM. OPTIMAL, an occupational therapy led self-management support programme for people with multimorbidity in primary care: a randomized controlled trial. BMC Family Practice 2015;16:59.

For peer review only - http://bmjopen.bmj.com/site/about/guidelines.xhtml

10. Giltin LN, Hauck WW, Dennis MP, Winter L, Hodgson N, Schinfeld S. Long-term effect on mortality of a home intervention that reduces functional difficulties in older adults: Results from a randomized trial. Journal of the American Geriatrics Society 2009;57(3):476-81.

11. Gitlin LN, Winter L, Dennis MP, Corcoran M, Schinfeld S, Hauck WW. A randomized trial of a multicomponent home intervention to reduce functional difficulties in older adults. Journal of the American Geriatrics Society 2006;54(5):809-16

12. Charlson ME, Pompei P, Ales KL, MacKenzie R. A new method of classifying prognostic comorbidity in longitudinal studies: development and validation. J Chronic Dis. 1987;40:373–383.

13. Greenfield S, Apolone G, McNeil B, Cleary P. The importance of coexistent disease in the occurrence of postoperative complications and one year recovery in patients undergoing total hip replacement: comorbidity and outcomes after hip replacement. Med Care. 1993;31:141–154.

14. Diederichs C, Berger K, Bartels DB. The Measurement of Multiple Chronic Diseases—A Systematic Review on Existing Multimorbidity Indices. J Gerontol A Biol Sci Med Sci. 2011 March;66A(3):301–311

15. Fairall, L., Petersen, I., Zani, B., Folb, N., Georgeu-Pepper, D., Selohilwe, O., Petrus, R., Mntambo, N., Bhana, A., Lombard, C. and Bachmann, M., 2018. Collaborative care for the detection and management of depression among adults receiving antiretroviral therapy in South Africa: study protocol for the CobALT randomised controlled trial. Trials, 19(1), p.193.

16. Petersen, I., Bhana, A., Folb, N., Thornicroft, G., Zani, B., Selohilwe, O., Petrus, R., Mntambo, N., Georgeu-Pepper, D., Kathree, T. and Lund, C., 2018. Collaborative care for the detection and management of depression among adults with hypertension in South Africa: study protocol for the PRIME-SA randomised controlled trial. Trials, 19(1), p.192.

17. Menezes, P., Quayle, J., Claro, H.G., da Silva, S., Brandt, L.R., Diez-Canseco, F., Miranda, J.J., Price, L.N., Mohr, D.C. and Araya, R., 2019. Use of a Mobile Phone App to Treat Depression Comorbid With Hypertension or Diabetes: A Pilot Study in Brazil and Peru. JMIR mental health, 6(4), p.e11698.

18. Gray, W.K., Richardson, J., McGuire, J., Dewhurst, F., Elder, V., Weeks, J., Walker, R.W. and Dotchin, C.L., 2016. Frailty screening in low-and middle-income countries: A systematic review. Journal of the American Geriatrics Society, 64(4), pp.806-823.

19. Fried LP, Tangen CM, Walston J et al. Frailty in older adults: Evidence for a phenotype. J Gerontol A Biol Sci Med Sci 2001;56A:M146–M156

For peer review only - http://bmjopen.bmj.com/site/about/guidelines.xhtml

20. van Boven JFM, van de Hei SJ, Sadatsafavi M. Making sense of cost-effectiveness analyses in respiratory medicine: a practical guide for non-health economists. Eur Respir J. 2019;53:3

21. Elliot Marseille, Bruce Larson, Dhruv S Kazi, James G Kahnd and Sydney Rosen. Thresholds for the cost–effectiveness of interventions: alternative approaches. Bull World Health Organ 2015;93:118–124

22. Arokiasamy et al. The impact of multimorbidity on adult physical and mental health in low and middle income countries: what does the study on global ageing and adult health (SAGE) reveal? BMC Medicine (2015) 13:178

23. Lee JT, Hamid F, Pati S, Atun R, Millett C (2015) Impact of Noncommunicable Disease Multimorbidity on Healthcare Utilisation and Out-OfPocket Expenditures in Middle-Income Countries: Cross Sectional Analysis. PLoS ONE 10(7): e0127199.doi:10.1371/journal.pone.0127199

24. Cookson R, Asaria M, Ali S, Ferguson B, Fleetcroft R, Goddard M, et al. Health Equity Indicators for the English NHS: a longitudinal whole-population study at the small-area level. Health Serv Deliv Res 2016;4(26).

25. Rijken M, Hujala A, van Ginneken E, Melchiorre MG, Groenewegen P, Schellevis F. Managing multimorbidity: Profiles of integrated care approaches targeting people with multiple chronic conditions in Europe. Health Policy. 2018 Jan;122(1):44-52.

26. Boerma T, Eozenou P, Evans D, Evans T, Kieny MP, Wagstaff A. Monitoring progress towards universal health coverage at country and global levels. PLoS Med. 2014;11(9):e1001731. Published 2014 Sep 22. doi:10.1371/journal.pmed.1001731

27. GBD 2015 Healthcare Access and Quality Collaborators. Healthcare Access and Quality Index based on mortality from causes amenable to personal health care in 195 countries and territories, 1990-2015: a novel analysis from the Global Burden of Disease Study 2015. Lancet. 2017 Jul 15;390(10091):231-266

28. Mair FS, May CR. Thinking about the burden of treatment. BMJ : British Medical Journal 2014; 349: g6680.

29. Leppin AL, Montori VM, Gionfriddo MR. Minimally Disruptive Medicine: A Pragmatically Comprehensive Model for Delivering Care to Patients with Multiple Chronic Conditions. Healthcare (Basel, Switzerland) 2015; 3(1): 50-63.

For peer review only - http://bmjopen.bmj.com/site/about/guidelines.xhtml

30. Eton DT, Ridgeway JL, Egginton JS, et al. Finalizing a measurement framework for the burden of treatment in complex patients with chronic conditions. Patient related outcome measures 2015; 6: 117-26.

31. Eton DT, Yost KJ, Lai J-s, et al. Development and validation of the Patient Experience with Treatment and Self-management (PETS): a patient-reported measure of treatment burden. Quality of Life Research 2017; 26(2): 489-503.

32. Tran V-T, Montori VM, Eton DT, Baruch D, Falissard B, Ravaud P. Development and description of measurement properties of an instrument to assess treatment burden among patients with multiple chronic conditions. BMC Medicine 2012; 10(1): 68.

33. Déruaz-Luyet A, Goran AA, Tandjung R, et al. Multimorbidity in primary care: protocol of a national cross-sectional study in Switzerland. BMJ Open 2015; 5(10): e009165.

34. Tran V-T, Messou E, Mama Djima M, Ravaud P, Ekouevi DK. Patients' perspectives on how to decrease the burden of treatment: a qualitative study of HIV care in sub-Saharan Africa. BMJ Quality & Safety 2019; 28(4): 266.

35. Duncan P, Murphy M, Man M-S, Chaplin K, Gaunt D, Salisbury C. Development and validation of the Multimorbidity Treatment Burden Questionnaire (MTBQ). BMJ Open 2018; 8(4): e019413.

36. Boyd CM, Wolff JL, Giovannetti E, et al. Healthcare task difficulty among older adults with multimorbidity. Medical care 2014; 52 Suppl 3: S118-25.

37. Gibbons CJ, Kenning C, Coventry PA, et al. Development of a Multimorbidity Illness Perceptions Scale (MULTIPleS). PLOS ONE 2013; 8(12): e81852.

38. Matima R, Murphy K, Levitt NS, BeLue R, Oni T. A qualitative study on the experiences and perspectives of public sector patients in Cape Town in managing the workload of demands of HIV and type 2 diabetes multimorbidity. PLoS One. 2018;13(3):e0194191. Published 2018 Mar 14. doi:10.1371/journal.pone.0194191

39. Naska A, Lagiou A, Lagiou P. Dietary assessment methods in epidemiological research: current state of the art and future prospects. F1000Res. 2017 Jun 16;6:926.

40. Shim JS, Oh K, Kim HC. Dietary assessment methods in epidemiologic studies. Epidemiol Health. 2014 Jul 22;36:e2014009.

41. de Oliveira Otto MC, Anderson CAM, Dearborn JL, Ferranti EP, Mozaffarian D, Rao G, Wylie-Rosett J, Lichtenstein AH; American Heart Association Behavioral Change for Improving Health Factors Committee of the Council on Lifestyle and Cardiometabolic Health and Council on Epidemiology and Prevention; Council on Cardiovascular and Stroke Nursing; Council on

For peer review only - http://bmjopen.bmj.com/site/about/guidelines.xhtml

Clinical Cardiology; and Stroke Council. Dietary Diversity: Implications for Obesity Prevention in Adult Populations: A Science Advisory From the American Heart Association. Circulation. 2018 Sep 11;138(11):e160-e168.

42. Rathnayake KM, Madushani P, Silva K. Use of dietary diversity score as a proxy indicator of nutrient adequacy of rural elderly people in Sri Lanka. BMC Res Notes. 2012 Aug 29;5:469.

43. Walls HL, Johnston D, Mazalale J, Chirwa EW. Why we are still failing to measure the nutrition transition. BMJ Glob Health. 2018 Feb 21;3(1):e000657.

44. Barr AL, Young EH, Sandhu MS. Objective measurement of physical activity: improving the evidence base to address non-communicable diseases in Africa. BMJ Glob Health. 2018 Oct 8;3(5):e001044.

45. Koyanagi A, Stubbs B, Vancampfort D. Correlates of sedentary behavior in the general population: A cross-sectional study using nationally representative data from six low- and middle-income countries. PLoS One. 2018 Aug 10;13(8):e0202222.

46. Vancampfort D, Stubbs B, Koyanagi A. Physical chronic conditions, multimorbidity and sedentary behavior amongst middle-aged and older adults in six low- and middle-income countries. Int J Behav Nutr Phys Act. 2017 Oct 27;14(1):147.

47. STEPwise approach to surveillance – available at: https://www.who.int/ncds/surveillance/steps/en/ last accessed 1st August 2019.

48. Chow CK, Lock K, Madhavan M, Corsi DJ, Gilmore AB, Subramanian SV, Li W, Swaminathan S, Lopez-Jaramillo P, Avezum A, Lear SA, Dagenais G, Teo K, McKee M, Yusuf S. Environmental Profile of a Community's Health (EPOCH): an instrument to measure environmental determinants of cardiovascular health in five countries. PLoS One. 2010 Dec 10;5(12):e14294.

49. Corsi DJ, Subramanian SV, McKee M, Li W, Swaminathan S, Lopez-Jaramillo P, Avezum A, Lear SA, Dagenais G, Rangarajan S, Teo K, Yusuf S, Chow CK. Environmental Profile of a Community's Health (EPOCH): an ecometric assessment of measures of the community environment based on individual perception. PLoS One. 2012;7(9):e44410

50. Kim S, Adamson KC, Balfanz DR, Brownson RC, Wiecha JL, Shepard D, Alles WF. Development of the Community Healthy Living Index: a tool to foster healthy environments for the prevention of obesity and chronic disease. Prev Med. 2010 Jan;50 Suppl 1:S80-5.

51. Hur MH. Demographic and Socioeconomic Determinants of Self-Efficacy: An Empirical Study of Korean Older Adults. Int J Aging Hum Dev. 2018;87(3):289-308.

For peer review only - http://bmjopen.bmj.com/site/about/guidelines.xhtml

52. Callander EJ, Schofield DJ. Impact of multidimensional poverty on the self-efficacy of older people: Results from an Australian longitudinal study. Geriatr Gerontol Int. 2017;17(2):308-14.

53. Kollia N, Caballero FF, Sanchez-Niubo A, Tyrovolas S, Ayuso-Mateos JL, Haro JM, et al. Social determinants, health status and 10-year mortality among 10,906 older adults from the English longitudinal study of ageing: the ATHLOS project. BMC Public Health. 2018;18(1):1357.

54. Qian H, Yuan C. Factors associated with self-care self-efficacy among gastric and colorectal cancer patients. Cancer Nurs. 2012;35(3):E22-31.

55. Schnell-Hoehn KN, Naimark BJ, Tate RB. Determinants of self-care behaviors in community-dwelling patients with heart failure. J Cardiovasc Nurs. 2009;24(1):40-7.

56. Baheiraei A, Bakouei F, Mohammadi E, Montazeri A, Hosseni M. The Social Determinants of Health in Association with Women's Health Status of Reproductive Age: A Population-Based Study. Iran J Public Health. 2015;44(1):119-29.

57. Walker RJ, Gebregziabher M, Martin-Harris B, Egede LE. Independent effects of socioeconomic and psychological social determinants of health on self-care and outcomes in Type 2 diabetes. Gen Hosp Psychiatry. 2014;36(6):662-8.

58. Caprara GV, Vecchione M, Alessandri G, Gerbino M, Barbaranelli C. The contribution of personality traits and self-efficacy beliefs to academic achievement: a longitudinal study. Br J Educ Psychol. 2011;81(Pt 1):78-96

59. Di Giunta L, Eisenberg N, Kupfer A, Steca P, Tramontano C, Caprara GV. Assessing Perceived Empathic and Social Self-Efficacy Across Countries. Eur J Psychol Assess. 2010;26(2):77-86.

60. van der Slot WM, Nieuwenhuijsen C, van den Berg-Emons RJ, Wensink-Boonstra AE, Stam HJ, Roebroeck ME. Participation and health-related quality of life in adults with spastic bilateral cerebral palsy and the role of self-efficacy. J Rehabil Med. 2010;42(6):528-35.

61. Liebke L, Bungert M, Thome J, Hauschild S, Gescher DM, Schmahl C, et al. Loneliness, social networks, and social functioning in borderline personality disorder. Personal Disord. 2017;8(4):349-56.

Contributorship

The concept of the paper arose from discussion at the GACD Multi-Morbidity Working Group. Individual sections of the manuscript were drafted by the named authors, coordinated by JRH, with all contributors providing important intellectual content and approving this final version for submission.

For peer review only

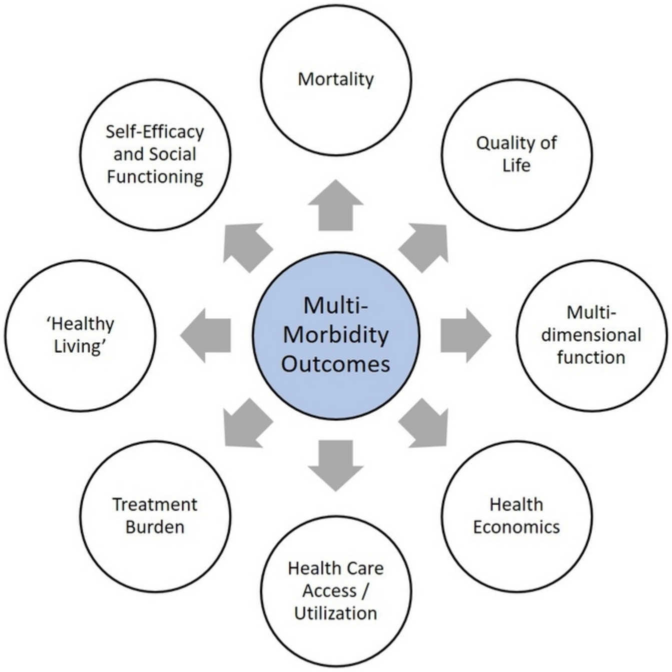

FIGURE 1: Eight domains of outcome measures for multi-morbidity interventions in LMIC.

84x56mm (300 x 300 DPI)

BMJ Open

A Critical Review of Multi-Morbidity Outcome Measures suitable for Low- and Middle Income Country Settings: perspectives from the Global Alliance for Chronic Diseases (GACD) Researchers

Journal:	*BMJ Open*
Manuscript ID	bmjopen-2020-037079.R1
Article Type:	Original research
Date Submitted by the Author:	28-Apr-2020
Complete List of Authors:	Hurst, John; UCL Medical School,, Academic Unit of Respiratory Medicine<br>Agarwal, Gina; McMaster University, Family Medicine<br>van Boven, Job F. M.; Univ Groningen<br>Daivadanam, Meena; Karolinska Institutet, Department of Public Health Sciences; Uppsala University , Department of Food, Nutrition and Dietetics<br>Gould, Gillian; The University of Newcastle, School of Medicine and Public Health; University of Newcastle Hunter Medical Research Institute,<br>Wan-Chun Huang, Erick ; Woolcock Institute of Medical Research, Sydney, Australia<br>Maulik, Pallab; The George Institute for Global Health,<br>Miranda, J. Jaime; Universidad Peruana Cayetano Heredia, CRONICAS Centre of Excellence in Chronic Diseases<br>Owolabi, M. O.; University of Ibadan College of Medicine, Medicine<br>Premji, Shahirose ; York University,<br>Soriano, Joan; Universidad Autónoma de Madrid,<br>Vedanthan, Rajesh; NYU Langone Health,<br>Yan, Lijing; Global Health Research Center, Duke Kunshan University, Kunshan, Jiangsu, China<br>Levitt, Naomi; University of Cape Town , medicine
**Primary Subject Heading**:	Global health
Secondary Subject Heading:	Cardiovascular medicine, Diabetes and endocrinology, Evidence based practice, General practice / Family practice, Global health
Keywords:	PRIMARY CARE, PUBLIC HEALTH, STATISTICS & RESEARCH METHODS, Clinical trials < THERAPEUTICS

SCHOLARONE™
Manuscripts

I, the Submitting Author has the right to grant and does grant on behalf of all authors of the Work (as defined in the below author licence), an exclusive licence and/or a non-exclusive licence for contributions from authors who are: i) UK Crown employees; ii) where BMJ has agreed a CC-BY licence shall apply, and/or iii) in accordance with the terms applicable for US Federal Government officers or employees acting as part of their official duties; on a worldwide, perpetual, irrevocable, royalty-free basis to BMJ Publishing Group Ltd ("BMJ") its licensees and where the relevant Journal is co-owned by BMJ to the co-owners of the Journal, to publish the Work in this journal and any other BMJ products and to exploit all rights, as set out in our licence.

The Submitting Author accepts and understands that any supply made under these terms is made by BMJ to the Submitting Author unless you are acting as an employee on behalf of your employer or a postgraduate student of an affiliated institution which is paying any applicable article publishing charge ("APC") for Open Access articles. Where the Submitting Author wishes to make the Work available on an Open Access basis (and intends to pay the relevant APC), the terms of reuse of such Open Access shall be governed by a Creative Commons licence – details of these licences and which Creative Commons licence will apply to this Work are set out in our licence referred to above.

Other than as permitted in any relevant BMJ Author's Self Archiving Policies, I confirm this Work has not been accepted for publication elsewhere, is not being considered for publication elsewhere and does not duplicate material already published. I confirm all authors consent to publication of this Work and authorise the granting of this licence.

For peer review only

A Critical Review of Multi-Morbidity Outcome Measures suitable for Low- and Middle Income Country Settings: perspectives from the Global Alliance for Chronic Diseases (GACD) Researchers

John R Hurst, Gina Agarwal, Job F M van Boven, Meena Daivadanam, Gillian S Gould, Erick Wan-Chun Huang, Pallab K Maulik, J Jaime Miranda, Mayowa O Owolabi, Shahirose Sadrudin Premji, Joan B Soriano, Rajesh Vedanthan, Lijing L Yan, Naomi S Levitt*

***: on behalf of the GACD Multi-Morbidity Working Group contributors:**
Ricardo Araya, Kirsten Bobrow, Niels H Chavannes, F Xavier Gómez-Olivé, Shabbar Jaffar, Bruce J Kirenga, Rianne M J J van der Kleij, Muralidhar M Kulkarni, Laura Loli-Dano, Patricio Lopez-Jaramillo, Shane Norris, Josefien van Olmen, Gary Parker, Trishul Siddharthan, Kamran Siddiqi, Najma Siddiqi, Antigona C Trofor

Corresponding Author:
John R Hurst
Professor of Respiratory Medicine
UCL Respiratory
University College London
London
UK

j.hurst@ucl.ac.uk

Funding
The article was supported by the Global Alliance for Chronic Diseases (GACD) secretariat. The Authors are investigators on individual studies funded in collaboration with the GACD.

Conflict of Interest
The Authors have no conflicts of interest to declare in relation to this work.

KEYWORDS: Multimorbidity; Outcome Assessment, Health Care;

WORDCOUNT: 4672

For peer review only

Abstract

OBJECTIVES: There is growing recognition around the importance of multi-morbidity in low- and middle-income country (LMIC) settings, and specifically the need for pragmatic intervention studies to reduce the risk of developing multi-morbidity, and of mitigating the complications and progression of multi-morbidity in LMICs. One of many challenges in completing such research has been the selection of appropriate outcomes measures. A 2018 Delphi exercise to develop a core-outcome set for multi-morbidity research (COSmm) did not specifically address the challenges of multi-morbidity in LMICs where the global burden is greatest, patterns of disease often differ and health systems are frequently fragmented. We therefore aimed to summarise and critically review outcome measures suitable for studies investigating mitigation of multi-morbidity in low- and middle-income country (LMIC) settings.

SETTING: LMIC.

PARTICIPANTS: people with multi-morbidity.

OUTCOME MEASURES: identification of all outcome measures.

RESULTS: We present a critical review of outcome measures across eight domains: mortality, quality of life, function, health economics, health-care access and utilization, treatment burden, measures of 'healthy living', and self-efficacy and social functioning.

CONCLUSIONS: Studies in multi-morbidity are necessarily diverse and thus different outcome measures will be appropriate for different study designs. Presenting the diversity of outcome measures across domains should provide a useful summary for researchers, encourage the use of multiple domains in multi-morbidity research, and provoke debate and progress in the field.

TRIAL REGISTRATION: Not applicable.

Strengths and Limitations

- There is no existing review of outcome measures suitable for use in studies to mitigate multi-morbidity in LMIC settings.
- The article is the written by the Global Alliance for Chronic Diseases researchers.
- It is not a systematic review.
- Further work is required to develop a core-outcome set for use in LMIC.

For peer review only

For peer review only - http://bmjopen.bmj.com/site/about/guidelines.xhtml

Introduction

There is growing recognition around the importance of multi-morbidity in low- and middle-income countries (LMICs) [1]. Multi-morbidity, as defined by the United Kingdom Academy of Medical Sciences (AMS) refers to "*the co-existence of two or more chronic conditions, each of which is either a physical non-communicable disease of long duration, a mental health condition of long duration, or an infectious disease of long duration*" [1]. The AMS report highlights challenges in delivering multi-morbidity research [2], including the selection of appropriate outcome measures. In 2018, Smith completed a Delphi exercise to develop a core-outcomes set for multi-morbidity research (COSmm) [3]. The highest scoring outcomes were health-related quality of life, mental health outcomes and mortality. Whilst ground-breaking, this process did not specifically target the challenges of multi-morbidity in LMICs where the global burden is greatest, patterns of disease often differ and health systems are frequently fragmented.

The Global Alliance for Chronic Diseases (GACD) is an alliance of health-research funders, whose research teams form a network of multidisciplinary researchers from both LMICs and high-income countries (HICs). We aim to reduce the impact of non-communicable diseases (NCDs) through a focus on implementation science research in LMICs, and high-priority populations in HICs. Recognizing synergies across our disease-specific programmes, in 2017 we formed a Multi-Morbidity Working Group and published a GACD Researchers' Statement concluding that "*a greater focus on multi-morbidity is overdue and necessary to successfully improve global health outcomes*", thus acknowledging the specific challenge of multi-morbidity in the LMIC context [4]. The statement went on to propose three strategic objectives, one of which was to change the way research is commissioned, funded and delivered when considering NCDs in LMICs.

Discussion with research funders subsequently highlighted that one barrier to funding research addressing multi-morbidity in LMICs was a perceived lack of robust outcome measures. We have therefore developed this GACD Researchers' perspective on outcome measures suitable for studies of multi-morbidity in LMICs, taking into account the challenges of (routine) data collection, and patient-provider factors such as differences in interpreting social constructs and health literacy. The intent is to build on the COSmm work [3]. Derived from a common base of expertise in NCD implementation research in LMICs, we present a diversity of potential measures that can accommodate different aspects of impact in LMICs, ranging from individual level outcomes to health service and health system effects. This is not an attempt to provide a core outcome measures set. Rather, together, the potential outcome measures inform different evaluations of effectiveness and/or process for multi-morbidity. We present these as a useful resource for those designing and reviewing intervention studies for multi-morbidity in LMIC settings, and hope this initiative may promote harmonization across studies that will be essential to better map the impact of multi-morbidity in LMIC settings.

For peer review only - http://bmjopen.bmj.com/site/about/guidelines.xhtml

Method

Potential outcome measures suitable for studies of multi-morbidity in LMICs were collected through a survey among the GACD multi-morbidity working group, and distilled by the writing committee (the Authors) into categories through consensus discussion. All GACD researchers were invited to take part in the multi-morbidity working group and those expressing interest were then invited to provide suggestions for suitable outcome measures via free-text e-mail to the group leads. In total, 31 group members participated (listed as the Authors and Contributors), with representation from all WHO Regions except the Eastern Mediterranean. GACD researchers have considerable collective experience conducting implementation science trials in LMIC settings. All measures had to be suitable for use in multi-morbidity intervention studies in LMIC, either at the individual or the population level, and from an implementation science perspective. Criteria for suitability included ease of measurement (such as availability of data, ease of data collection, availability of local translations and cost), generalizability (applicability of the proposed outcome across diverse populations within and between LMIC settings) and statistical considerations (the feasability of demonstrating a clinically significant change with conventional statistical significance). Each outcome approach is fully described below. The initial synthesis was reviewed by members of the GACD Multi-Morbidity Working Group for additional comments and suggestions (the Contributors). The resulting narrative review summarizes the group's collective thoughts within each domain of outcome measures studied.

Patient and Public Involvement

Patients or the public were not involved in the design, conduct, reporting, or dissemination plans of our research.

Outcome Measures for Multi-Morbidity Interventions in LMIC

1. Mortality

Death is the final common outcome for all individuals. Thus (premature) mortality is the most broadly applicable, generalizable, and comparable outcome for multi-morbidity research. Indeed, mortality was considered as an "essential" core outcome measure for multi-morbidity research according to the COSmm consensus [3].

However, precisely because mortality is so broadly applicable, it suffers from a lack of specificity. While cause-specific mortality is a potential solution to the issue of specificity, this approach moves away from the goal of multi-morbidity-based outcome consideration. In addition, mortality does not reflect the quality of life that an individual experiences during the time of survival; particularly in the context of multi-morbidity, both disability and quality-of-life considerations are important in terms of an individual's experience of illness, wellness and life. Indeed, death is not always the most important outcome from a patient-centered perspective, as has been demonstrated in studies assessing patient preferences of different potential health outcomes [5-7] and conceptualized as Disability-Adjusted Life Years.

Practical challenges with mortality as an outcome measure include statistical power and sample size for an outcome that is relatively rare compared to other outcomes and proxies, potentially

For peer review only - http://bmjopen.bmj.com/site/about/guidelines.xhtml

requiring much longer follow-up periods, except for older and/or more severely affected populations. It is, however, generally easy to measure and while the primary cause may be ascertained through techniques such as verbal autopsy (2016 WHO VA standard) [8], assessing the contribution of multi-morbidity at verbal autopsy is more challenging. Whilst misclassifying the cause of death can impact the effect size for cause-specific mortality, power will be preserved for all-cause mortality. In some LMICs, ascertainment of deaths remains difficult due to the lack of mature vital registry systems and cultural traditions promoting deaths at home with delay in reporting.

Thus, mortality as an outcome for multi-morbidity research has been infrequently utilized, particularly in the context of LMIC settings [9-11]. Demographic surveillance sites that have a long record of verbal autopsy could, however, provide a useful data reservoir to examine associations between multi-morbidity and mortality.

2. Generic Quality of Life scales

Health-related quality of life (HRQoL) instruments measure multidimensional wellbeing and functioning. Such scales may be generic such as EQ-5D and SF-36, or disease (/area) specific. While disease-specific measures may have better content and face validity as well as better responsiveness and sensitivity to change compared to generic measures, generic measures are (by definition) not disease specific and likely better for comparison of HRQoL among different diseases and for diseases in combination, an important consideration for multi-morbidity research. Tools to assess the related construct of self-reported well-being have been reviewed and summarized elsewhere [12].

Among generic tools, the COSmm consensus [3] ranked the EQ-5D, SF36 and ’12, and Global quality of life (WHOQOL-BREF) most highly.

The EQ-5D [13] has been widely used since introduction in the 1990s, facilitating health-economic analysis (see below). It is designed to be completed by the participant and is available in multiple languages and thus widely applicable. The EQ-5D questionnaire has two components (health-state description and evaluation). In the health state description, health status is measured across five dimensions: mobility, self-care, usual activities, pain/discomfort, and anxiety/depression. In the evaluation section, the respondents evaluate their overall health status using a visual analogue scale.

The SF-36 [14] has 36 questions across eight domains: vitality, physical functioning, bodily pain, general health perceptions, physical role functioning, emotional role functioning, social role functioning and mental health.

The WHOQOL-BREF [15] is an abbreviated version of the WHOQOL-100 quality of life assessment, originally developed by the WHOQOL Group working across fifteen international field centers to develop a quality of life assessment applicable across multiple settings.

For peer review only - http://bmjopen.bmj.com/site/about/guidelines.xhtml

HRQoL tools have a number of advantages over mortality as an outcome, being amenable to changes in the short term. HRQoL outcomes are particularly meaningful as the aim of clinical treatment and management is generally optimizing quality of life. Consequently, managing multi-morbidity needs to take quality of life into account both as an outcome marker, but also an input factor into formulating clinical management. Practical considerations in LMIC include the availability of valid translations in local languages (these are more often available for the more commonly used tools, in the more commonly used languages, but coverage remains incomplete), and the challenges of use in populations with low literacy or understanding of visual-analogue scales. Other unanswered questions include whether thresholds for minimum clinically important differences on these scales should be altered in the context of multi-morbidity. Notably, some common NCDs such as hypertension are not generally associated with significant symptom burden.

For peer review only

3. Multi-dimensional indices of function

The AMS [1] recommended that reports of multi-morbidity should provide details of functional deficits, or disabilities and frailty. In both instances the recommendation was made that this should be coded using a standardized classification scheme. For the former, the WHO Disability Assessment Schedule 2.0 (WHODAS 2.0) or the International Classification of Functioning, Disability and Health (ICF) were suggested. For the latter, the cumulative deficit model of frailty or Fried's phenotype model was recommended (see below).

WHO Disability Assessment Schedule

The WHODAS 2.0 has been widely used in epidemiological and observational studies in LMICs. It is a self-administered 12 item questionnaire that assesses six different adult life tasks over the preceding month. The specific areas covered are 1) understanding and communication; 2) self-care; 3) mobility; 4) interpersonal relationships; 5) work and household roles and 6) community and civic roles. WHODAS has been included as a secondary outcome measure in three multi-morbidity trials in LMIC (currently unreported [16, 17]).

Frailty assessment instruments:

There are many methods to assess frailty including the Fried Index, the Frailty Index and the British Frailty Index. While these have been used to examine the prevalence, correlates or outcomes of frailty in LMIC, further validation is still required in these settings [18]. Of the various metrics, the Fried Index [19] has been the most commonly used in LMIC. This index measures frailty by the presence of three or more of five physical deficits - exhaustion, weakness, slowness, low levels of activity and weight loss. Three of the items are collected using questionnaires, but slowness is assessed using a walking test and weakness by assessing grip strength. The Frailty Index has also been commonly used in LMICs and uses the presence or absence of medical conditions or poor performance on functional tasks to assess the number of deficits present and thus frailty [18]. Using frailty as an outcome measure for intervention studies in patients with multi-morbidity in LMIC may be limited by factors such as a lack of equipment (for example, to measure grip strength), and the question remains as to how susceptible to change such measurements are, and what a minimum clinically important

For peer review only - http://bmjopen.bmj.com/site/about/guidelines.xhtml

difference (MCID) might be. Despite this, frailty instruments remain an important outcome in LMIC settings given that frailty may be a confounding factor in self-care, treatment adherence and family burden.

Assessment of physical functioning:
Physical functioning measures are commonly studied outcomes. The most frequently used indices include activities of daily living (such as eating, dressing and toileting), instrumental activities of daily living (such as shopping and answering phone calls), and the Barthel Index (self-reported outcomes on degree of assistance needed for mobility, self-care and continence). Smith [3] described activities of daily living, physical function and physical activity as core outcomes in multi-morbidity interventions. For ADL the following measures received greatest support: Frenchay Activities Index (FAI), Nottingham Extended Activities of Daily Living (NEADL) and the Instructions for Activities of Daily Living questionnaire (ADL/ IADL), but these have not been evaluated in the context of LMICs.

The modified Rankin Scale is an example of a disease-specific (in this case, stroke) composite outcome measure including rating of functioning from no interference with daily life, through various degrees of disability to death. These outcomes are relatively easy to assess and have particular relevance in LMICs as people generally express strong desires in maintaining physical functioning including their ability to work, avoiding financial consequences and burden on family caregiving.

4. Health Economic implications
The AMS report [1] highlighted the economic burden of multi-morbidity in LMICs and thus health economic implications are relevant in any consideration of multi-morbidity outcome measures. However, most economic data on multi-morbidity were gathered in HICs and the question arises as to whether measurement instruments, data and outcomes commonly used to assess cost implications of multi-morbidity in HICs are applicable to LMIC settings.

One of the most common economic evaluations of healthcare interventions makes use of a technique called cost-effectiveness analysis and specifically the *incremental cost-effectiveness ratio* (ICER) [20]. The method to calculate the ICER is not disease specific, making it just as suitable to assess multi-morbidity interventions as single disease interventions. However, it requires specific attention to the definitions and collection of costs and effect data in LMICs. Within this ratio, costs and effects can be defined, measured, and calculated in different ways, of which some are more suitable in economic assessment of multi-morbidity interventions in LMICs than others. Interpretation of the ratio may differ in different settings.

In healthcare, interventions can impact different types of direct and indirect costs within and outside healthcare systems. The different costs to be included in cost-effectiveness analysis depends on the perspective that is taken (e.g. the healthcare payer, the society, the patient, or the family). Costs that directly result from the intervention and which occur within healthcare systems should be included when a healthcare payer perspective is taken. However, in LMICs

For peer review only - http://bmjopen.bmj.com/site/about/guidelines.xhtml

that lack universal health coverage, the perspective of the patient and family may be more relevant and a key focus could be on out-of-pocket costs. Examples of indirect costs are work productivity losses and these costs are especially relevant when a patient or societal perspective is taken.

In health economic studies, the effect of intervention uses a measure that is independent of a specific disease: the quality-adjusted life year (QALY). The QALY is a combination of utility (preferably measured using the EQ-5D) and survival. With the EQ-5D, certain health states are defined, to which a specific utility is assigned. Utility is the value a society gives to a specified health state and for each country a specific algorithm should be estimated from large general population samples. In many LMIC settings these still need to be further developed to allow for generalizable models of effectiveness.

While most HICs have defined guidelines and make use of fixed thresholds or ranges to assess whether a certain ICER is considered cost-effective, such guidelines and thresholds are generally lacking in LMICs. This complicates the interpretation of cost-effectiveness analyses in LMICs. As a general rule, the World Health Organization (WHO) defines an intervention that costs less than three times the gross domestic product per capita as cost-effective [21]. It is important to note that the implications of economic analyses discussed here are not challenges specific to multi-morbidity, but are nonetheless suitable for the study of multi-morbidity.

5. Health Care Access and Utilization

Multi-morbidity is associated with repeated care seeking, often at different providers. This not only results in multiple interactions with health care settings through outpatient and inpatient admissions but also involves para-medical services and practitioners of traditional medicine.

Although we identified no study that has specifically looked at generating or testing multi-morbidity related healthcare access indices in LMICs, the WHO Study on Global Ageing and Adult Health (SAGE) which focused on LMICs tracked indicators specific to multi-morbidity in ageing populations [22, 23]. These included the number of outpatient visits in the last 12 months, overnight hospital stays in the past three years, and the number of overnight stays in hospital in the past 12 months. A UK National Health Service document [24] outlines equity indicators that may also map multi-morbidity relevant in LMIC settings, and some of these have direct healthcare access relevance such as emergency hospitalizations for chronic conditions and repeat emergency hospitalizations in the same year. Access to medicines listed on the WHO Essential Medications list would provide another metric, as would recommendations on attention to comorbidity and pharmacological interactions in treatment guidelines.

This lack of LMIC specific multi-morbidity indices to plot healthcare access leads to a critically important avenue of research that could draw on that conducted in HICs [25]. The latter work lists a range of objectives that need to be addressed in healthcare practices catering to clients with multi-morbidity and lists a set of preventive services for such cases which health facilities should provide. Health-seeking behaviour is a further dimension related to healthcare access

For peer review only - http://bmjopen.bmj.com/site/about/guidelines.xhtml

that is shaped by unique socioeconomic and cultural contexts faced by patients in LMICs. We suggest it would be useful to develop health-seeking behaviour indices relevant across LMICs. This needs a contextual framework to best understand what is feasible and what can be tracked within specific LMIC settings, acknowledging the challenges introduced by the fragmentation of care and the multiplicity of levels of provision of care in the public and private sectors. Such indices could be linked with existing monitoring frameworks used to assess Universal Health Coverage [26].

The Global Burden of Disease initiative has recently incorporated a new metric at national level termed the Healthcare Access and Quality (HAQ) Index [27]. The HAQ index is a scale from 0 to 100, calculated by measuring mortality rates from causes that should not be fatal (amenable mortality) in the presence of effective medical care. This correlates with the Socio-demographic Index, a measure of overall development consisting of income *per capita*, average years of education, and total fertility rates.

6. Treatment burden

The burden of treatment, a relatively new concept, emerged from disease-centered healthcare systems in response to the growing needs of coping with chronic conditions. In the context of multi-morbidity, this may be considered as the workload and impact on a patient as a result of receiving medical care [28]. High treatment burden may lead to overwhelmed patients who struggle to access healthcare and adhere to suggested treatment whilst coordinating their own care and other aspects of life, a particular issue among patients with multi-morbidity. As a consequence, polypharmacy and non-adherence to treatment and poor clinical outcomes may follow, resulting in an even higher burden of treatment, a deterioration cycle depicted in the Cumulative Complexity Model [29]. Therefore, assessing treatment burden is a priority in order to achieve better quality healthcare, and treatment burden is a potential outcome measure in interventions directed against multi-morbidity. There is also the challenge, more pronounced in LMICs, that in areas of no care there can be no 'burden' from treatment which it is impossible to access.

Assessing the burden of treatment is not an easy task. It generally requires multi-dimensional measures that are tailored to the medical condition(s), health system(s) and cultural background. Tailoring to specific conditions may diminish value in multi-morbidity. Eton proposed a conceptual framework of treatment burden based on qualitative inquiries to patients with chronic conditions, consisting of three themes and 15 subthemes [30]. A number of tools for evaluating treatment burden for patients with multi-morbidity have been developed in the past few years. Eton designed and validated the Patient Experience with Treatment and Self-management (PETS) [31]. The Treatment Burden Questionnaire (TBQ) is another instrument, consisting of 15 items [32] and later further adapted [33, 34]. In 2018, Duncan published the Multimorbidity Treatment Burden Questionnaire (MTBQ), a ten-item measure initially validated in primary care in the United Kingdom [35]. The Healthcare Task Difficulty (HCTD) questionnaire is an 11-question tool designed to measure only one aspect – perceived difficulty in performing healthcare management tasks [36]. Finally, the Multi-morbidity Illness

For peer review only - http://bmjopen.bmj.com/site/about/guidelines.xhtml

Perceptions Scale (MULTIPLes), unlike other instruments, was designed to measure the perceived impact of multi-morbidity [37]. The scale includes treatment burden (six questions) as one of the subscales.

As these questionnaires are relatively new, validation and translation for different populations and geographic areas remain limited, especially in LMICs. Exploring the notion and measurement of treatment burden in LMIC remains relatively unexplored [34, 38], as does the important concept of patient-reported experience measures in LMIC settings which may themselves affect health outcomes [39].

There are a number of remaining issues to be considered before applying these tools in LMICs. Firstly, the strengths and limitations of each tool should be examined as careful validation has often not been conducted in such settings. Secondly, using mixed methods incorporating experiences and opinions from patients and healthcare providers may help identify relevant issues relating to differences in contexts, cultures and health system structures. Thirdly, as all of these instruments have been available for less than a decade, longitudinal evidence of change over time is absent.

7. Measures of 'Healthy Living'

Multi-morbidity is complex to operationalize, which makes common denominators very relevant. Measures of 'Healthy Living' are direct common denominators for being at risk of developing individual components of multi-morbidity, and thus measuring change in these measures provides potential generic outcomes of interventions to mitigate future multi-morbidity. Most current behavioral interventions have targeted only one behaviour at a time.

Healthy living encompasses many different aspects of health and wellbeing, including diet, physical activity including sedentary behaviour, tobacco and alcohol consumption, developing health literacy, maintaining good hygiene, and sanitation. Most current behavioral interventions have targeted only one behaviour at a time.

Diet: Dietary assessments are complex. Self-reported dietary intake measurements are the most common form of dietary assessments, which include prospective recording of actual food consumed or retrospective recall [40, 41]. With respect to multi-morbidity, the focus must be on long-term usual intake. Dietary diversity scores are one such measure that can be estimated for the individual, or the household using counts of food items (food variety score) or food groups (dietary diversity score) consumed over a pre-specified period [42, 43]. Dietary diversity can be estimated at the Household level using the Household Dietary Diversity Score (HDDS), which assesses household access to a variety of foods, or at individual level for women and children respectively using the Minimum Dietary Diversity for Women of Reproductive Age tool (MDD-W) and WHO Infant and Young Child Minimum Dietary Diversity Tool (IYCF-MDD) [44].

Physical activity (PA) including sedentary behaviour: Convincing interventional evidence showing a clear dose-response relationship between PA and improved health outcomes comes

For peer review only - http://bmjopen.bmj.com/site/about/guidelines.xhtml

mainly from HICs, although associations of PA with reduced cardiovascular mortality and morbidity are available globally [45]. Sedentary behaviour, defined as those that involve sitting or reclining and low levels of energy expenditure during waking hours [46], has also been associated with having at least two morbidities, independent of light, moderate or vigorous PA [46, 47] in HICs and LMICs. The Global Physical Activity Questionnaire (GPAQ) that is part of the WHO STEPwise Approach to Chronic Disease Risk Factor Surveillance data collection tool [48] is a commonly used tool to collect self-reported data on PA. The GPAQ which is a shorter (16-item) version of the longer International Physical Activity Questionnaire (IPAQ) also assesses sitting time in addition to PA in three domains (work, travel and leisure-time). This is used to estimate the duration of moderate to vigorous physical activity (MVPA) or intensity in terms of MET (metabolic equivalent)-minutes per week of total and domain-specific activities. However, agreement between PA estimated by GPAQ and more objective measures has been moderate at best. Objective measures of PA allow real-time monitoring and can be easily completed using an application on a mobile device or a wearable pedometer or accelerometer, although this has mostly been tested in HIC settings. Considering the rapid acceleration of smart phone ownership in LMIC, and the availability of cheaper but robust wearable devices, these are now viable options and an optional tool to capture objective PA has since been incorporated into the GPAQ.

Tobacco and alcohol use: Tobacco use has been consistently linked as a causative factor for chronic respiratory disorders such as chronic obstructive pulmonary disease, cardiovascular disease and many cancers including lung cancer. Similarly, alcohol use has strong associations with NCDs. Ever and current use of tobacco or current use of alcohol are commonly used assessments in addition to questions focusing on frequency and amount of consumption, and these are part of the WHO STEPS instrument [48]. Where available, verification of smoking status can be achieved through measurement of carbon monoxide or urinary cotinine. Assessment of household, environmental and occupational airborne exposures are more complex.

Healthy living index: In addition to individual risks and behaviors, composite indicators that assess healthy living may be more relevant in the context of multi-morbidity. Tools to assess the environment in terms of its potential to offer opportunities for healthy living have been limited, especially in LMICs. Environmental Profile of a Community's Health (EPOCH) is a quantitative tool designed to capture community perceptions of tobacco, nutrition, and social environments, validated in five countries (China, India, Brazil, Colombia, and Canada) [49, 50]. EPOCH comprises an objective assessment of the physical environment, and an interviewer-administered questionnaire on residents' perceptions of their community to capture both objective and subjective measures of the environment [49]. The Community Healthy Living Index (CHLI), developed in the US assessed the environmental support potential of a community across five domains assessing a specific venue: schools, afterschool child care sites, work sites, neighborhoods, and communities-at-large [51]. Such tools could be adapted for use in LMICs.

For peer review only - http://bmjopen.bmj.com/site/about/guidelines.xhtml

8. Self-efficacy and social functioning

Self-efficacy and social functioning relate to social determinants of health such as age, gender, marital status, family background, employment, education level and socioeconomic status [52-58], affecting in turn how an individual is able to look after their health conditions (self-efficacy) and interact in society with other individuals leading a fulfilling life (social functioning). This raises the important question of whether indices of self-efficacy and social functioning may be suitable as outcomes measures in studies to mitigate multi-morbidity in LMIC settings.

There are limited studies that explore which social determinants are more influential than others in determining self-efficacy and social functioning. Positive personality traits and higher self-esteem demonstrated in adolescence positively affect self-efficacy [59]. Competent behaviour, such as skills of focusing on others' well-being, affiliative behaviours/interpersonal cooperation and participation, which are culturally valued and socially competent are associated with higher self-efficacy [60, 61]. Liebke and colleagues [62] reported that loneliness and social functioning are associated. Loneliness may be caused by impaired social skills, such as maintaining conversations or expressing feelings, which are essential to adequate social functioning [62]. Values placed on social determinants of health may vary across different cultures. Differences in cultural traditions may affect the sources of self-efficacy belief systems [52, 55, 56].

Given the multitude of cultural factors affecting the precursors of self-efficacy and social functioning, populations in LMICs may have fewer opportunities to develop such skills. Therefore, whilst measures of self-efficacy and social functioning could be used as multi-morbidity outcome measure in LMIC, a single index is unlikely to be useful across all settings.

Conclusions

The case has been made for the growing global importance of multi-morbidity, the need for pragmatic intervention studies to reduce the risk of developing multi-morbidity in LMIC settings, and of mitigating the complications and progression of multi-morbidity. One of many challenges in such research has been the selection of appropriate outcomes measures.

We present the GACD Researchers' perspective on outcome measures suitable for multi-morbidity intervention studies in the context of LMICs. We have considered outcome measures across eight domains (Figure 1). Some represent direct measurements of clinical outcomes, whilst others represent intermediate variables on the pathway to multi-morbidity. Some measures are single, others are composite. They vary in their ease of collection and cost. It is critical to choose appropriate outcomes for the study design, cultural context and participant preference in order to demonstrate and understand the effect of an intervention, and our aim is therefore not to suggest a preference of one outcome measure over any other. Studies in multi-morbidity are necessarily diverse and thus different outcome measures will be appropriate for different study designs. As with the COSmm consensus [3], we recognize the key importance of mortality and health-related quality of life as multi-morbidity outcomes, and these are suitable for use in LMIC settings. Many other outcomes from the COSmm work, including patient-reported impacts and behaviors (such as treatment burden and self-efficacy), physical activity

For peer review only - http://bmjopen.bmj.com/site/about/guidelines.xhtml

and function, and health systems indicators (notably health economic indices) are also suitable for LMIC settings, though in the context and with the caveats that we have described above. Some of the challenges applying these outcome measures in LMIC are also relevant in HIC.

The diversity of outcome measures across domains demonstrated here should provide a useful summary for researchers, and encourage the use of multiple domains in multi-morbidity research, rather than just a single outcome measure. Ultimately, the proof of utility for these outcome measures will be the demonstration that an effective multi-morbidity intervention can improve the health of the community in which it is tested. Meanwhile, there remains the urgent need for further study and development of outcome measures suitable for multi-morbidity intervention studies in the context of LMIC.

There are limitations to this work, which is not intended to be a core outcome set, nor a systematic review. Development of both these would be an important contribution to the field, as would further work to understand the perceptions of these outcome measures from people directly affected by multi-morbidity and tools suitable for assessing patient-reported experience in the context of multi-morbidity. Here, we present a critical, narrative synthesis describing the range of outcome measures that might be selected for use in such settings, and their challenges. The key strength of our work is the broad representation of views from GACD researchers who have considerable collective experiene of implementation science research in LMIC settings. We anticipate this will be useful to other researchers designing and conducting such studies, and to provoke debate and progress in the field.

Figure Legend

FIGURE 1: *Eight domains of outcome measures for multi-morbidity interventions in LMIC.*

Data Availability

No additional data available.

For peer review only - http://bmjopen.bmj.com/site/about/guidelines.xhtml

References

1. Academy of Medical Sciences. Multimorbidity: a priority for global health research: Full report. Available at https://acmedsci.ac.uk/policy/policy-projects/multimorbidity last accessed 26th April 2020.

2. Academy of Medical Sciences. Advancing research to tackle multimorbidity: the UK and LMIC perspectives workshop report. Available at https://acmedsci.ac.uk/policy/policy-projects/multimorbidity last accessed 26th April 2020.

3. Smith SM, Wallace E, Salisbury C, Sasseville M, Bayliss E, Fortin M. A Core Outcome Set for Multimorbidity Research (COSmm). Ann Fam Med. 2018 Mar;16(2):132-138.

4. Hurst JR, Dickhaus J, Maulik PK, Miranda JJ, Pastakia SD, Soriano JB, Siddharthan T, Vedanthan R; GACD Multi-Morbidity Working Group. Global Alliance for Chronic Disease researchers' statement on multimorbidity. Lancet Glob Health. 2018 Dec;6(12):e1270-e1271.

5. Vaanholt MCW, Kok MM, von Birgelen C, Weernink MGM, van Til JA. Are component endpoints equal? A preference study into the practice of composite endpoints in clinical trials. Health Expect. 2018 Dec;21(6):1046-1055. doi: 10.1111/hex.12798. Epub 2018 Aug 14. PubMed PMID: 30109764; PubMed Central PMCID: PMC6250862.

6. van Summeren JJ, Schuling J, Haaijer-Ruskamp FM, Denig P. Outcome prioritisation tool for medication review in older patients with multimorbidity: a pilot study in general practice. Br J Gen Pract. 2017 Jul;67(660):e501-e506. doi: 10.3399/bjgp17X690485. Epub 2017 Mar 27. PubMed PMID: 28347987; PubMed Central PMCID: PMC5565860.

7. Fried TR, Tinetti M, Agostini J, Iannone L, Towle V. Health outcome prioritization to elicit preferences of older persons with multiple health conditions. Patient Educ Couns. 2011 May;83(2):278-82. doi: 10.1016/j.pec.2010.04.032. Epub 2010 May 31. PubMed PMID: 20570078; PubMed Central PMCID: PMC2945432.

8. WHO. Verbal autopsy standards: ascertaining and attributing causes of death. Available at https://www.who.int/healthinfo/statistics/verbalautopsystandards/en/ last accessed 26th April 2020.

9. Garvey J, Connolly D, Boland F, Smith SM. OPTIMAL, an occupational therapy led self-management support programme for people with multimorbidity in primary care: a randomized controlled trial. BMC Family Practice 2015;16:59.

10. Giltin LN, Hauck WW, Dennis MP, Winter L, Hodgson N, Schinfeld S. Long-term effect on mortality of a home intervention that reduces functional difficulties in older adults: Results from a randomized trial. Journal of the American Geriatrics Society 2009;57(3):476-81.

For peer review only - http://bmjopen.bmj.com/site/about/guidelines.xhtml

11. Gitlin LN, Winter L, Dennis MP, Corcoran M, Schinfeld S, Hauck WW. A randomized trial of a multicomponent home intervention to reduce functional difficulties in older adults. Journal of the American Geriatrics Society 2006;54(5):809-16.

12. Linton MJ, Dieppe P, Medina-Lara A. Review of 99 self-report measures for assessing well-being in adults: exploring dimensions of well-being and developments over time. BMJ Open. 2016;6(7):e010641.

13. Dolan P. Modeling valuations for EuroQol health states. Med Care. 1997;35(11):1095–1108.

14. Jenkinson C, Coulter A, Wright L. Short form 36 (SF36) health survey questionnaire: normative data for adults of working age. BMJ. 1993;306(6890):1437–1440. doi:10.1136/bmj.306.6890.1437.

15. Skevington SM, Lotfy M, O'Connell KA; WHOQOL Group. The World Health Organization's WHOQOL-BREF quality of life assessment: psychometric properties and results of the international field trial. A report from the WHOQOL group. Qual Life Res. 2004;13(2):299–310. doi:10.1023/B:QURE.0000018486.91360.00.

16. Fairall L, Petersen I, Zani B, Folb N, Georgeu-Pepper D, Selohilwe O, Petrus R, Mntambo N, Bhana A, Lombard C and Bachmann M. Collaborative care for the detection and management of depression among adults receiving antiretroviral therapy in South Africa: study protocol for the CobALT randomised controlled trial. Trials 2018;19(1), p.193.

17. Petersen I, Bhana A, Folb N, Thornicroft G, Zani B, Selohilwe O, Petrus R, Mntambo N, Georgeu-Pepper D, Kathree T and Lund C. Collaborative care for the detection and management of depression among adults with hypertension in South Africa: study protocol for the PRIME-SA randomised controlled trial. Trials 2018;19(1), p.192.

18. Gray WK, Richardson J, McGuire J, Dewhurst F, Elder V, Weeks J, Walker RW, Dotchin CL. Frailty screening in low-and middle-income countries: A systematic review. Journal of the American Geriatrics Society 2016;64(4), pp.806-823.

19. Fried LP, Tangen CM, Walston J et al. Frailty in older adults: Evidence for a phenotype. J Gerontol A Biol Sci Med Sci 2001;56A:M146–M156.

20. van Boven JFM, van de Hei SJ, Sadatsafavi M. Making sense of cost-effectiveness analyses in respiratory medicine: a practical guide for non-health economists. Eur Respir J. 2019;53:3.

21. Marseille E, Larson B, Kazi DS, Kahnd JG, Rosen S. Thresholds for the cost–effectiveness of interventions: alternative approaches. Bull World Health Organ 2015;93:118–124.

For peer review only - http://bmjopen.bmj.com/site/about/guidelines.xhtml

22. Arokiasamy *et al*. The impact of multimorbidity on adult physical and mental health in low and middle income countries: what does the study on global ageing and adult health (SAGE) reveal? BMC Medicine (2015) 13:178.

23. Lee JT, Hamid F, Pati S, Atun R, Millett C (2015) Impact of Noncommunicable Disease Multimorbidity on Healthcare Utilisation and Out-OfPocket Expenditures in Middle-Income Countries: Cross Sectional Analysis. PLoS ONE 10(7): e0127199.doi:10.1371/journal.pone.0127199.

24. Cookson R, Asaria M, Ali S, Ferguson B, Fleetcroft R, Goddard M, et al. Health Equity Indicators for the English NHS: a longitudinal whole-population study at the small-area level. Health Serv Deliv Res 2016;4(26).

25. Rijken M, Hujala A, van Ginneken E, Melchiorre MG, Groenewegen P, Schellevis F. Managing multimorbidity: Profiles of integrated care approaches targeting people with multiple chronic conditions in Europe. Health Policy. 2018 Jan;122(1):44-52.

26. Boerma T, Eozenou P, Evans D, Evans T, Kieny MP, Wagstaff A. Monitoring progress towards universal health coverage at country and global levels. PLoS Med. 2014;11(9):e1001731. Published 2014 Sep 22. doi:10.1371/journal.pmed.1001731.

27. GBD 2015 Healthcare Access and Quality Collaborators. Healthcare Access and Quality Index based on mortality from causes amenable to personal health care in 195 countries and territories, 1990-2015: a novel analysis from the Global Burden of Disease Study 2015. Lancet. 2017 Jul 15;390(10091):231-266.

28. Mair FS, May CR. Thinking about the burden of treatment. BMJ : British Medical Journal 2014; 349: g6680.

29. Leppin AL, Montori VM, Gionfriddo MR. Minimally Disruptive Medicine: A Pragmatically Comprehensive Model for Delivering Care to Patients with Multiple Chronic Conditions. Healthcare (Basel, Switzerland) 2015; 3(1): 50-63.

30. Eton DT, Ridgeway JL, Egginton JS, et al. Finalizing a measurement framework for the burden of treatment in complex patients with chronic conditions. Patient related outcome measures 2015; 6: 117-26.

31. Eton DT, Yost KJ, Lai J-s, et al. Development and validation of the Patient Experience with Treatment and Self-management (PETS): a patient-reported measure of treatment burden. Quality of Life Research 2017; 26(2): 489-503.

32. Tran V-T, Montori VM, Eton DT, Baruch D, Falissard B, Ravaud P. Development and description of measurement properties of an instrument to assess treatment burden among patients with multiple chronic conditions. BMC Medicine 2012; 10(1): 68.

For peer review only - http://bmjopen.bmj.com/site/about/guidelines.xhtml

33. Déruaz-Luyet A, Goran AA, Tandjung R, et al. Multimorbidity in primary care: protocol of a national cross-sectional study in Switzerland. BMJ Open 2015; 5(10): e009165.

34. Tran V-T, Messou E, Mama Djima M, Ravaud P, Ekouevi DK. Patients' perspectives on how to decrease the burden of treatment: a qualitative study of HIV care in sub-Saharan Africa. BMJ Quality & Safety 2019; 28(4): 266.

35. Duncan P, Murphy M, Man M-S, Chaplin K, Gaunt D, Salisbury C. Development and validation of the Multimorbidity Treatment Burden Questionnaire (MTBQ). BMJ Open 2018; 8(4): e019413.

36. Boyd CM, Wolff JL, Giovannetti E, et al. Healthcare task difficulty among older adults with multimorbidity. Medical care 2014; 52 Suppl 3: S118-25.

37. Gibbons CJ, Kenning C, Coventry PA, et al. Development of a Multimorbidity Illness Perceptions Scale (MULTIPleS). PLOS ONE 2013; 8(12): e81852.

38. Matima R, Murphy K, Levitt NS, BeLue R, Oni T. A qualitative study on the experiences and perspectives of public sector patients in Cape Town in managing the workload of demands of HIV and type 2 diabetes multimorbidity. PLoS One. 2018;13(3):e0194191. Published 2018 Mar 14. doi:10.1371/journal.pone.0194191.

39. Birkhäuer J, Gaab J, Kossowsky J, et al. Trust in the health care professional and health outcome: A meta-analysis. PLoS One. 2017;12(2):e0170988.

40. Naska A, Lagiou A, Lagiou P. Dietary assessment methods in epidemiological research: current state of the art and future prospects. F1000Res. 2017 Jun 16;6:926.

41. Shim JS, Oh K, Kim HC. Dietary assessment methods in epidemiologic studies. Epidemiol Health. 2014 Jul 22;36:e2014009.

42. de Oliveira Otto MC, Anderson CAM, Dearborn JL, Ferranti EP, Mozaffarian D, Rao G, Wylie-Rosett J, Lichtenstein AH; American Heart Association Behavioral Change for Improving Health Factors Committee of the Council on Lifestyle and Cardiometabolic Health and Council on Epidemiology and Prevention; Council on Cardiovascular and Stroke Nursing; Council on Clinical Cardiology; and Stroke Council. Dietary Diversity: Implications for Obesity Prevention in Adult Populations: A Science Advisory From the American Heart Association. Circulation. 2018 Sep 11;138(11):e160-e168.

43. Rathnayake KM, Madushani P, Silva K. Use of dietary diversity score as a proxy indicator of nutrient adequacy of rural elderly people in Sri Lanka. BMC Res Notes. 2012 Aug 29;5:469.

For peer review only

For peer review only - http://bmjopen.bmj.com/site/about/guidelines.xhtml

44. Walls HL, Johnston D, Mazalale J, Chirwa EW. Why we are still failing to measure the nutrition transition. BMJ Glob Health. 2018 Feb 21;3(1):e000657.

45. Barr AL, Young EH, Sandhu MS. Objective measurement of physical activity: improving the evidence base to address non-communicable diseases in Africa. BMJ Glob Health. 2018 Oct 8;3(5):e001044.

46. Koyanagi A, Stubbs B, Vancampfort D. Correlates of sedentary behavior in the general population: A cross-sectional study using nationally representative data from six low- and middle-income countries. PLoS One. 2018 Aug 10;13(8):e0202222.

47. Vancampfort D, Stubbs B, Koyanagi A. Physical chronic conditions, multimorbidity and sedentary behavior amongst middle-aged and older adults in six low- and middle-income countries. Int J Behav Nutr Phys Act. 2017 Oct 27;14(1):147.

48. STEPwise approach to surveillance – available at: https://www.who.int/ncds/surveillance/steps/en/ last accessed 26th April 2020.

49. Chow CK, Lock K, Madhavan M, Corsi DJ, Gilmore AB, Subramanian SV, Li W, Swaminathan S, Lopez-Jaramillo P, Avezum A, Lear SA, Dagenais G, Teo K, McKee M, Yusuf S. Environmental Profile of a Community's Health (EPOCH): an instrument to measure environmental determinants of cardiovascular health in five countries. PLoS One. 2010 Dec 10;5(12):e14294.

50. Corsi DJ, Subramanian SV, McKee M, Li W, Swaminathan S, Lopez-Jaramillo P, Avezum A, Lear SA, Dagenais G, Rangarajan S, Teo K, Yusuf S, Chow CK. Environmental Profile of a Community's Health (EPOCH): an ecometric assessment of measures of the community environment based on individual perception. PLoS One. 2012;7(9):e44410.

51. Kim S, Adamson KC, Balfanz DR, Brownson RC, Wiecha JL, Shepard D, Alles WF. Development of the Community Healthy Living Index: a tool to foster healthy environments for the prevention of obesity and chronic disease. Prev Med. 2010 Jan;50 Suppl 1:S80-5.

52. Hur MH. Demographic and Socioeconomic Determinants of Self-Efficacy: An Empirical Study of Korean Older Adults. Int J Aging Hum Dev. 2018;87(3):289-308.

53. Callander EJ, Schofield DJ. Impact of multidimensional poverty on the self-efficacy of older people: Results from an Australian longitudinal study. Geriatr Gerontol Int. 2017;17(2):308-14.

54. Kollia N, Caballero FF, Sanchez-Niubo A, Tyrovolas S, Ayuso-Mateos JL, Haro JM, et al. Social determinants, health status and 10-year mortality among 10,906 older adults from the English longitudinal study of ageing: the ATHLOS project. BMC Public Health. 2018;18(1):1357.

For peer review only - http://bmjopen.bmj.com/site/about/guidelines.xhtml

55. Qian H, Yuan C. Factors associated with self-care self-efficacy among gastric and colorectal cancer patients. Cancer Nurs. 2012;35(3):E22-31.

56. Schnell-Hoehn KN, Naimark BJ, Tate RB. Determinants of self-care behaviors in community-dwelling patients with heart failure. J Cardiovasc Nurs. 2009;24(1):40-7.

57. Baheiraei A, Bakouei F, Mohammadi E, Montazeri A, Hosseni M. The Social Determinants of Health in Association with Women's Health Status of Reproductive Age: A Population-Based Study. Iran J Public Health. 2015;44(1):119-29.

58. Walker RJ, Gebregziabher M, Martin-Harris B, Egede LE. Independent effects of socioeconomic and psychological social determinants of health on self-care and outcomes in Type 2 diabetes. Gen Hosp Psychiatry. 2014;36(6):662-8.

59. Caprara GV, Vecchione M, Alessandri G, Gerbino M, Barbaranelli C. The contribution of personality traits and self-efficacy beliefs to academic achievement: a longitudinal study. Br J Educ Psychol. 2011;81(Pt 1):78-96.

60. Di Giunta L, Eisenberg N, Kupfer A, Steca P, Tramontano C, Caprara GV. Assessing Perceived Empathic and Social Self-Efficacy Across Countries. Eur J Psychol Assess. 2010;26(2):77-86.

61. van der Slot WM, Nieuwenhuijsen C, van den Berg-Emons RJ, Wensink-Boonstra AE, Stam HJ, Roebroeck ME. Participation and health-related quality of life in adults with spastic bilateral cerebral palsy and the role of self-efficacy. J Rehabil Med. 2010;42(6):528-35.

62. Liebke L, Bungert M, Thome J, Hauschild S, Gescher DM, Schmahl C, et al. Loneliness, social networks, and social functioning in borderline personality disorder. Personal Disord. 2017;8(4):349-56.

Contributorship

The concept for this paper arose from discussion at the GACD Multi-Morbidity Working Group chaired by JRH. The authors (JRH, GA, JFMvB, MD, GSG, EW-CH, PKM, JJM, MOO, SSP, JBS, RV, LLY and NSL) planned and conducted the e-mail survey. All authors and contributors (RA, KB, NHS, FXG-O, SJ, BJK, RMJJvdK, MMK, LL-D, PL-J, SN, JvO, GP, TS, KS, NS, ACT) provided suggestions for outcome measures. Individual sections of the manuscript were drafted by the authors, coordinated by JRH into a first complete draft. All authors revised this initial draft. All authors and contributors provided important intellectual content on the revised draft and approved the final version for submission.

For peer review only - http://bmjopen.bmj.com/site/about/guidelines.xhtml

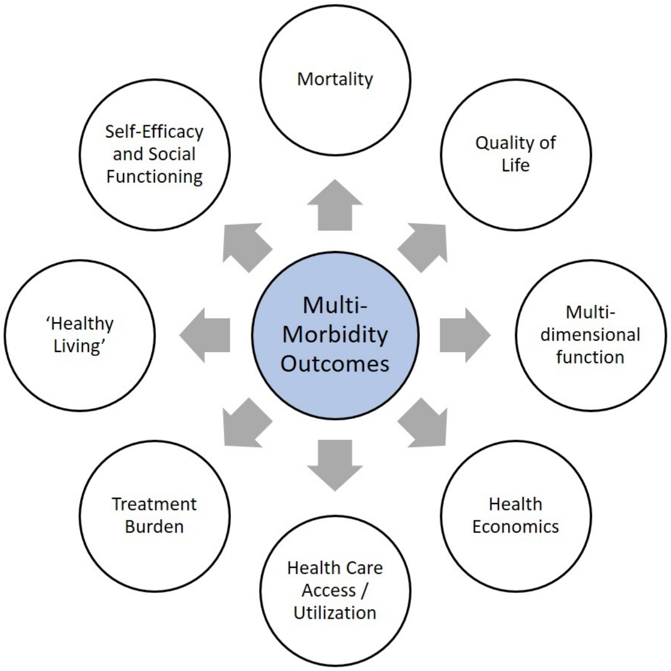

FIGURE 1: Eight domains of outcome measures for multi-morbidity interventions in LMIC.

84x56mm (400 x 400 DPI)
